# Teens and social media: determinants of problematic social media use among adolescents in Poland – risks and protective factors

**DOI:** 10.13075/ijomeh.1896.02835

**Published:** 2026

**Authors:** Katarzyna Porwit, Monika Szkultecka-Dębek, Zbigniew Izdebski

**Affiliations:** 1 University of Warsaw, Centre of Migration Research, Warsaw, Poland; 2 University of Social Sciences, Faculty of Applied Sciences, Warsaw, Poland; 3 University of Warsaw, Faculty of Education, Warsaw, Poland; 4 University of Zielona Gora, Collegium Medicum, Department of Humanization of Medicine and Sexology, Zielona Góra, Poland; 5 University of Warsaw, Faculty of Medicine, Warsaw, Poland

**Keywords:** Poland, adolescents, protective factors, social media addiction, cross-sectional studies, health risk behaviors

## Abstract

**Objectives:**

The study aimed to investigate the determinants of problematic social media use (PSMU) among Polish adolescents by accounting for time spent on social media, sociodemographic characteristics, individual resources, social environment, and health factors, and to compare the findings with previous research.

**Material and Methods:**

A cross-sectional, anonymous, online survey was conducted in 2024. The study was performed among adolescents in the Lubuskie Voivodeship, Poland and included 9073 students aged 12–19 years (mean [M] ± standard deviation [SD]15.16±1.56 years). Problematic social media use was assessed using the 9-item *Social Media Disorder Scale* (SMDS). Independent variables included sociodemographic characteristics, time spent on social media, health literacy, future orientation, school achievements, subjective health complaints, well-being, school stress, parent and peer relations, and school climate, measured with validated instruments derived mainly from the Health Behaviour in School-aged Children (HBSC) survey protocol and the KIDSCREEN-27 questionnaire.

**Results:**

Problematic social media use was identified in 12.7% of participants. Higher PSMU was observed among younger adolescents. The strongest predictor was the time spent on social media >5 h/day. Poorer health and well-being – including multiple psychosomatic complaints, lower well-being, and higher school stress – were strongly associated with PSMU. Lower future orientation, low health literacy, and poorer relationships with parents increased the likelihood of PSMU, whereas older age, higher future orientation, and supportive parent relationships decreased.

**Conclusions:**

Multiple factors influenced PSMU among adolescents, although the overall model fit remained modest. Boys were more likely to experience PSMU, representing a novel observation. Higher future orientation, supportive relationships with parents, and older age were key protective factors. A non-linear relationship was found between PSMU and peer support as well as school climate.

## Highlights

- Time spent on social media was the strongest predictor of problematic social media use (PSMU).
- Higher future orientation and good parent relations were main protecting factors.
- Multiple health complaints were also a risk factor strongly associated with PSMU.
- In the final model the higher likelihood of PSMU was among boys.

## INTRODUCTION

The growing availability of smartphones and mobile internet has led to a surge in the popularity of online platforms and messaging apps. The period 2010–2012 can be described as the era of the full globalisation of social media. Significantly, today's adolescents have grown up entirely within a digitally saturated environment. Unlike previous generations, contemporary youth are exposed to and engage with social media from an exceptionally early age. Thus, navigating digital platforms has become an inseparable dimension of their everyday reality [1].

Problematic social media use (PSMU) is a pattern of social media use that is passive, excessive or difficult to control and that leads to significant negative consequences in daily functioning [2]. Sometimes it is described as a form of behaviour resembling addiction [3]. As a relatively new phenomenon, it can also be considered through a variety of other theoretical approaches [4].

Previous studies, depending on the scales, sample, country and year, estimate the share of PSMU at 5–31% [5]. Teenage populations generally exhibit higher rates of PSMU compared to adults; some analyses indicate rates of up to approx. 35% in adolescent samples [3]. In the Health Behaviour in School-aged Children survey (HBSC), the proportion of pupils reporting problematic media use increased from 7.6% in 2018 [6] to 11.5% in 2022 [7]. Media consumption among adolescents intensified particularly during the COVID-19 pandemic [8]. Furthermore, results from government research indicate that PSMU occurs among Polish adolescents at a rate of 11–15% within the 8-year-old, 12-year-old and 16-year-old age groups [9].

The recent rise in PSMU has raised significant concerns about its potential impacts on young people [10]. Research has demonstrated a strong association between PSMU and higher levels of depressive symptoms, anxiety, and stress, along with lower mental and social well-being [3], as well as higher levels of substance use compared to non-problematic users [10]. Both social anxiety and loneliness have been identified as risk factors for PSMU [2]. Previous studies have shown that addictive social media use is associated with reduced life satisfaction [11]. Intensive social media use disrupts cognitive functioning by lowering concentration, which ultimately leads to poorer academic performance [10].

Although this topic has received considerable attention in scientific literature for a decade and has been studied in Poland [12–14] there are no current Polish studies conducted in the local post-pandemic context.

The current study examined the determinants of PSMU among Polish adolescents. Consequently, several hypotheses (H) were formulated regarding these relationships. Based on prior research, it was hypothesized that risk factors, such as time spent on social media and the severity of psychosomatic symptoms, would be positively associated with PSMU (H1). In contrast, individual resources, including health literacy (HL) and future orientation, as well as social resources, such as the quality of social relationships, were expected to be negatively associated with PSMU, serving as protective factors (H2). Additionally, it was postulated that the level of PSMU would vary by age and gender (H3), although these effects were treated as exploratory in nature.

## MATERIAL AND METHODS

### Participants

The survey was conducted in March–May 2024 via anonymous online platform among students in the Lubuskie Voivodeship, Poland. A total of 88 schools – 44 primary (3 in each district and 4 in each of the major cities) and 44 secondary (following the same distribution) were randomly selected from the Ministry of National Education's database. Schools were invited to administer the survey at least 1 class from 3 educational levels; grade 7 in primary schools (approx. 13 years old) as well as grade 9 and 11 in secondary schools (approx. 15 years old and 17 years old, respectively). Students completed the questionnaires at school after parental consent was obtained. The study was approved by the Institutional Ethics Committee of Collegium Medicum, University of Zielona Góra, Zielona Góra, Poland (protocol code RCM-CM-KBUZ.031.39.2023 approved on November 17, 2023) [15].

A total of 9411 questionnaires were considered valid. Only students who answered all 9 questions assessing PSMU were included in the analyses, resulting in a final analytical sample of 9073 participants.

The age of participants was mean (M) ± standard deviation (SD) 15.16±1.56 years (range 12.25–19.25 years) in the analyzed sample. Students from more senior grades were less numerous (in grade 7 – 40.7%, in grade 9 – 37.2%, in grade 11 – 22.1%). Girls constituted a larger share of the sample than boys (54.7% vs. 45.3%), and the proportion of girls increased across successive grades (51.5%, 56.6%, and 57.3%, respectively) (p< 0.001).

### Tools

The questionnaire used in the study was titled: *What Young People in Lubuskie Voivodeship Think and How Do They Live*. It was cross-sectional and consisted of 11 thematic blocks. A significant part of the questions was derived from the HBSC study protocol [16]. An additional module of the survey was the KIDSCREEN-27 questionnaire. A total of 58 questions were used for the analyses, creating 15 variables. The dependent variable was the problematic use of social media. In the analyses, the variables were divided into 5 groups: socio-demographic, individual resources, well-being, social environment and time spent on social media. The distribution of answers to individual questions or categorized indexes can be found in Tables 1 and 2.

**Table 1:** Problematic social media use (PSMU) and demographic characteristics and individual resources, in an online survey conducted among 9073 students from Lubuskie Voivodship, Poland, March–May 2024

Variable	Participants (N = 9073) [n (%)]	PSMU score (M±SD)	Problematic users [%]	χ^2^	p
Demographic		2.53±2.41	12.7		
gender				20.123	<0.001
boys	4104 (45.2)	2.21±2.44	10.9		
girls	4689 (54.8)	2.80±2.36	14.1		
school grade				41.150	<0.001
grade 7	3685 (40.6)	2.76±2.50	14.8		
grade 9	3384 (37.3)	2.53±2.39	12.5		
grade 11	2004 (22.1)	2.12±2.23	8.9		
place of residence				0.243	0.886
city	1956 (21.6)	2.49±2.44	13.0		
small town	3854 (42.4)	2.51±2.40	12.6		
village	3263 (36.0)	2.49±2.42	12.5		
family affluence				3.534	0.171
poor	3055 (34.1)	2.63±2.46	13.5		
average	4431 (49.5)	2.46±2.36	12.0		
affluent	1465 (16.4)	2.51±2.41	12.4		
Individual resources					
health literacy				142.308	<0.001
low	2258 (25.5)	3.18±2.68	19.9		
average	5608 (63.3)	2.37±2.25	10.4		
high	993 (11.2)	1.98±2.40	9.5		
future orientation				223.320	<0.001
low	2087 (23.5)	3.32±2.71	21.5		
average	4635 (52.2)	2.49±2.29	11.1		
high	2162 (24.3)	1.83±2.08	6.9		
school achievements				21.290	<0.001
average and below	4382 (48.5)	2.74±2.46	14.4		
good	3236 (35.9)	2.35±2.32	10.8		
very good	1412 (15.6)	2.32±2.43	11.4		

**Table 2:** Problematic social media use (PSMU) and health and well-being indicators, social environment and support, and declared time spent on social media, in an online survey conducted among 9073 students from Lubuskie Voivodship, Poland, March–May 2024

Variable	Participants (N = 9073) [n (%)]	PSMU score (M±SD)	Problematic users [%]	χ^2^	p
Well-being indicators					
HBSC-SCL				268.67	<0.001
0–1	3893 (42.9)	1.78±2.07	6.1		
2+	5180 (57.1)	3.01±2.49	17.6		
WHO-5				158.844	<0.001
good mood	3848 (43.2)	1.99±2.25	8.0		
reduced mood	2869 (32.3)	2.78±2.37	14.0		
at risk of depression	2185 (24.5)	3.18±2.54	19.0		
school stress				112.322	<0.001
low	2292 (25.5)	1.89±2.35	8.7		
average	4550 (50.7)	2.56±2.29	11.6		
high	2136 (23.8)	3.18±2.52	18.9		
Social environment and support					
relationships with parents				227.134	<0.001
poor	1815 (20.3)	3.43±2.66	22.4		
average	5256 (58.7)	2.47±2.31	11.3		
good	1976 (21.0)	1.79±2.13	6.7		
peers and social support				67.232	<0.001
low	2025 (22.6)	2.94±2.60	17.7		
average	5023 (56.1)	2.41±2.29	10.5		
high	1910 (21.3)	2.39±2.44	12.4		
school climate				65.168	<0.001
unfavorable	2442 (27.2)	2.97±2.53	17.2		
average	4263 (47.6)	2.45±2.29	10.8		
supportive	2263 (25.2)	2.19±2.42	10.9		
Declared time spent on social media				252.874	<0.001
0–1 h/day	2902 (32.1)	1.83±2.22	7.5		
2–4 h/day	4028 (44.6)	2.53±2.30	11.3		
≥5 h/day	2101 (23.3)	3.51±2.52	22.3		

HBSC-SCL – *HBSC Symptom Checklist*; PSMU – problematic social media use; WHO-5 – *World Health Organization-Five Well-Being Index*.

### Dependent variable

#### Problematic Social Media Use

Social media was defined as social networking sites, instant messengers and online meeting tools. In the survey the *Social Media Disorder Scale* (SMDS) adapted from the HBSC study [16] was used. Respondents answered 9 questions describing specific symptoms: preoccupation, tolerance, withdrawal, persistence, displacement, problem, deception, escape, and conflict. Responses were encoded as binary (0 – no, 1 – yes) and summed to obtain a PSMU score (range 0–9). As suggested by the authors of the scale, adolescents who reported >6 symptoms during the past year were categorized as PSMU. In the analysis of the results, records containing missing data in any of the 9 SMDS questions were excluded.

### Independent variables

#### Sociodemographic variables

Gender was assessed using 3 response options: boy, girl, or other. Respondents who indicated “other” were assigned a grammatical gender based on their chosen identity for the analysis of the results.

In addition to age and gender, sociodemographic variables included place of residence and family affluence. The *Family Affluence Scale* (FAS) is a tool developed and used in the HBSC study [16]. It comprises 6 questions, summed to produce a total score ranging 0–13 pts. In this article, families were divided into 3 categories: affluent (10–13 pts), average (7–9 pts) and poor (0–6 pts).

Regarding the place of residence, respondents could choose between a village, a smaller town (41 such locations in the voivodeship), or a city with >100 000 inhabitants (2 such locations in the voivodeship).

#### Time spent on social media

The variable was based on declaration how many hours a day a student spends on social media. For the purposes of the analysis, 9 possible answers (0 h, 0.5 h, 1 h, 2 h, 3 h, 4 h, 5 h, 6 h, and 7 h) were grouped into categories: 0–1 h, 2–4 h, and ≥5 h.

### Invidual resources

#### Health literacy

The short version of the *Health Literacy in School-aged Children* (HLSAC-5) scale was used, as adapted from the HBSC study [16]. The instrument consists of 5 items covering 5 theoretical components: theoretical knowledge, practical knowledge, critical thinking, self-awareness, and citizenship. Respondents were asked to rate to their agreement with each statement by selecting 1 of 4 response options, ranging from “not at all true” to “absolutely true.”

The summary index has a range of 5–20 pts. The results of HLSAC-5 were categorized as low (5–12 pts), average (13–17 pts), and high (18–20 pts). In the study sample scale was homogeneous, with the principal component explaining 57.14% of the total variance. Cronbach's α was 0.819, indicating good internal consistency. A total of 97.6% of respondents completed this scale.

#### Future orientation

To assess future orientation, the abbreviated index of the *Future Time Perspective* (FTP-5) was used based on the publicly available *Zimbardo Time Perspective Inventory* [17]. Participants were asked to determine on a Likert scale ranging from 1 (completely false) to 5 (completely true), how accurately statements regarding planning and the fulfilment of duties and goals described them.

The results were standardized to a range of 0–100 and categorized into 3 FTP levels: low (<40 pts), average (40–70 pts), and high (>70 pts). The FTP-5 scale was homogeneous, with the principal component explaining 40.54% of the total variance. Cronbach's α was 0.630, which is considered acceptable for a short scale. A total of 97.9% of respondents completed this scale.

#### School achievements

A subjective measure was used to assess school achievement by asking students the question from the HBSC survey [16]: “What do you think your teachers think of your academic performance and other achievements in school compared to other students in the class?” There were 4 response categories: very good, good, average, below average. For analysis, the responses were recoded into 3 categories: average or below, good, very good. A total of 99.5% of the sample answered this question.

### Health and well-being indicators

#### Subjective health complaints

The *HBSC Symptom Checklist* (HBSC-SCL), an 8-item scale [16], was used to assess psychosomatic complaints. Participants were asked how often they had experienced the following symptoms during the past 6 months: headaches, stomach aches, backaches, feeling low, irritability, nervousness, difficulties in falling asleep, and dizziness. Responses were recorded on a 5-point scale, ranging from “almost every day” to “never”. To interpret the results, a dichotomous variable, was created; young people who reported at least 2 symptoms more than once a week were classified as experiencing multiple health complaints, compared to those who experienced such symptoms less frequently. Psychometric analysis showed good scale reliability: Cronbach's α was 0.862 with 51.76% of the variance explained. Complete scale scores were obtained for 97.8% of the sample.

#### Well-being

The self-reported *World Health Organization-Five Well-Being Index* (WHO-5) [16] consists of 5 questions related to energy levels, positive mood, and general interests over the past 2 weeks. The results were standardized on a 0–100 scale. According to WHO international recommendations, the cut-off points were defined as follows: 0–28 pts – risk of depression; 29–50 pts – reduced mood; 51–100 pts – good mood. In the collected sample, the WHO-5 scale was homogeneous and showed very good reliability (Cronbach's α = 0.844), explaining 61.91% of the variance. All 5 items of the scale were answered by 98.1% of participants.

#### School stress

Three questions from the first module of the *Adolescent Stress Questionnaire* (ASQ) were incorporated into the study, as adapted in the HBSC research protocol [16]. The following 3 sources of school stress were evaluated: learning incomprehensible things, too high expectations of teachers, and the pressure to keep up with schoolwork. Adolescents rated their level of stress on a 5-point scale, ranging from 0 – “not stressful at all or there was no such situation” to 4 – “very stressful.” A total index ranging 0–12 pts was built. It was divided into 3 categories: low (0–3 pts), average (4–9 pts) and high (10–12 pts) school-related stress. Psychometric analysis indicated that the scale was homogeneous explaining 72.50% of variance and demonstrating good internal consistency (Cronbach α = 0.810). All scale questions were answered by 99.0% of respondents.

#### Social environment and support

Social relations were assessed using the KIDSCREEN-27 questionnaire, a widely used tool for measuring health-related quality of life, serving as a shortened version of the full KIDSCREEN-52 [18].

For the analyses 2 indexes from KIDSCREEN-27 were used: *Parent Relations* and *Peers and Social Support*. Participants were instructed to respond with reference to the previous week, indicating the frequency of various experiences on a 5-point scale (from 1 – never to 5 – always). Scores were standardized to a 0–100-point scale, and cut-off points were defined as follows: <40 – poor, 40–80 – average, >80 – good.

The school climate was assessed using 4 items relating to relationships in the school. Two questions concerned the perception of relationships between students and 2 addressed relationships with teachers (acceptance and trust). Responses were recorded on a 5-point scale ranging from “strongly disagree” to “strongly agree.” The items were combined to form a total score ranging 0–16 pts. Based on the scores spread, 3 interpretative categories of school climate were defined: 0–6 pts – unfavourable, 7–10 pts – average, 11–16 pts –favourable.

In the collected dataset, the KIDSCREEN-27 indices demonstrated homogeneity and acceptable or good reliability, with Cronbach's α coefficients of 0.692 (school climate), 0.868 (peer support) and 0.867 (parent relations). The proportion of missing data for the individual subscales ranged from 1.2%, 1.3% and 1.4%, respectively.

### Data analysis methods

The distributional characteristics of the dependent variable were examined for the entire sample. Mean values of the dependent variable (the summarised PSMU indicator) were calculated for subgroups based on blocks of selected variables: socio-demographic characteristics, individual resources, health and well-being indicators, social environment, and time spent on social media.

To verify the hypotheses, the first step involved assessing the proportion of problematic social media users in the sample. The significance of differences in the results was examined using the χ^2^ test.

To identify predictors of PSMU variability, a hierarchical logistic regression analysis was estimated for the entire sample. In the regression model, the dependent variable was dichotomous (0 – non-problematic, 1 – problematic). To verify the hypotheses, variables were entered into the model in blocks, based on theoretical and empirical considerations consistent with Tables 1 and 2 (demographic variables, time spent on media use, individual resources, followed by health and well-being indicators and the social environment in the final block). This approach allowed to assess the incremental contribution of each set of predictors to the overall model fit. Model improvement was evaluated using the change in Nagelkerke's R² between successive models.

## RESULTS

The score of the total PSMU in the sample is higher than the median (Me) value of 2.00 (M ± SD 2.53±2.41). The distribution of the variable is positively skewed and slightly asymmetric (skewness = 0.905, kurtosis = 0.084), and the Q–Q plot largely corresponds to the normal distribution, with minor deviations in the tails. The scale has a 1-factor structure explaining 38.25% of the variance and demonstrates good internal consistency (α = 0.793). The problematic users accounted for 12.7% of the sample.

### Determinants of PSMU

#### Univariate analysis

The mean values of the total PSMU score in relation to demographic characteristics, which were entered to control for basic participant attributes are presented in Table 1. Only gender and age (school grade) were associated with PSMU. The older the student, the lower the average PSMU score (2.76 for the youngest 7th-grade students and 2.12 for the oldest high school students). Similarly, the proportion of problematic users decreased with age (from 14.8% to 8.9%). Girls experienced the phenomenon significantly more strongly than boys (M = 2.80 vs. 2.21, 14.1% vs. 10.9%). Differences related to family wealth and place of residence were not statistically significant.

All examined individual resources showed a significant inverse relationship with PSMU (Table 1). Individuals with a high FTP level had the lowest average PSMU score (1.83) and the lowest proportion of problematic users (6.9), while among those with the lowest FTP intensity, problematic users occurred 3 times more often than in the group with the highest FTP (21.5%). The differences in PSMU depending on HL level were slightly smaller than in the case of FTP (9.5% of problematic users among people with high HL and 19.9% among those with low HL). A similar pattern was observed for school achievements: the higher the student's grades, the lower the intensity of PSMU, although here the differences between the groups were not as significant as in the case of the other 2 individual resource variables.

The examined health and wellness indicators were significantly associated with the examined dependent variable. Individuals reporting multiple complaints had a significantly higher mean PSMU score (3.01) than those reporting lower frequency of symptoms (1.78). Among individuals without multiple complaints the proportion of problematic users was significantly lower (6.1% vs. 17.6%). The lower the well-being WHO-5 index, the stronger the association with PSMU were observed. Individuals with a good mood had an average PSMU score of 1.99, while those at risk of depression had a score of 3.18. Among those with the lowest mood, there were 11 p.p. more problematic users than among those with the highest mood. Similarly higher levels of perceived school stress were associated with higher PSMU scores (M = 1.89–3.18).

The analysis showed significant differences in the severity of PSMU depending on the perceived social environment, particularly the quality of the relationship with parents. Individuals who felt that their parents did not treat them fairly and did not have time or attention for them had the highest mean PSMU scores (3.43) and the highest proportion of problematic users (22.4%), compared to students with average (2.47, 11.3%) and good relationships (1.79, 6.7%).

The relationship with peers was not linear. Compared to individuals with average relationships (2.41, 10.5%), both those with low and high peers support had higher mean PSMU scores (2.94, 17.7%), although the difference was smaller for the latter (2.39, 12.4%).

The more supportive school environment, the lower the PSMU scores (a decrease in the average value from 2.97 through 2.45 to 2.19). However, the proportion of problematic users was almost the same among individuals rating the school climate as average or good (10.8% and 10.9%, respectively).

The group that spent the most time on social media had the highest average PSMU scores (3.51 for individuals declaring ≥5 h/day vs. 1.83 for those using ≤1 h/day). In this group, the proportion of problematic users (22.3%) was even higher than among individuals with low FTP (Table 1), but slightly lower than among those with poor relationships with their parents (Table 2).

#### Multivariate analysis

The independent variables included in the model showed negligible collinearity (variance inflation factor [VIF] = 1.006–1.567). After introducing socio-demographic variables into the model (N = 8086), gender and age were significant predictors (Table 3). Girls were 35.5% more likely to exhibit PSMU. The older the students, the significantly lower the probability of occurrence of the phenomenon: grade 9 by 18.1% and grade 11 by 46.6% less than grade 7 students. The place of residence and family affluence (FAS) were not significant. Nagelkerke's R² for this model was very low (0.015).

**Table 3:** Hierarchical logistic regression model (blocks 1–4) of the determinants of problematic social media use (PSMU) (N = 8021), 2024, Poland

	B	SE	Wald	df	p	OR	95% CI OR
Block 1							
gender							
boys (ref.)							
girls	0.304	0.070	18.911	1	0.000	1.355	1.181–1.553
school grade							
grade 7 (ref.)			41.243	2	0.000		
grade 9	–0.200	0.075	7.140	1	0.008	0.819	0.707–0.948
grade 11	–0.628	0.098	41.058	1	0.000	0.534	0.441–0.647
place of residence							
city (ref.)			0.229	2	0.892		
small town	0.031	0.090	0.118	1	0.732	1.031	0.865–1.230
village	–0.003	0.093	0.001	1	0.972	0.997	0.831–1.196
family affluence							
poor (ref.)			2.661	2	0.264		
average	–0.106	0.075	2.003	1	0.157	0.899	0.776–1.042
affluent	–0.137	0.103	1.779	1	0.182	0.872	0.712–1.067
constant	–1.880	0.101	346.172	1	0.000	0.153	
Block 2							
gender							
boys (ref.)							
girls	0.025	0.074	0.118	1	0.732	1.026	0.887–1.185
school grade							
grade 7 (ref.)			52.130	2	0.000		
grade 9	–0.290	0.076	14.484	1	0.000	0.748	0.644–0.869
grade 11	–0.704	0.099	50.321	1	0.000	0.494	0.407–0.601
place of residence							
city (ref.)			0.311	2	0.856		
small town	0.036	0.091	0.157	1	0.692	1.037	0.867–1.239
village	–0.004	0.094	0.002	1	0.962	0.996	0.828–1.198
family affluence							
poor (ref.)			2.362	2	0.307		
average	–0.099	0.076	1.688	1	0.194	0.906	0.780–1.052
affluent	–0.135	0.104	1.675	1	0.196	0.874	0.712–1.072
declared time spent on social media							
0–1 h/day (ref.)			204.198	2	0.000		
2–4 h/day	0.486	0.095	25.998	1	0.000	1.626	1.349–1.960
≥5 h/day	1.319	0.099	176.027	1	0.000	3.739	3.077–4.543
constant	–2.289	0.116	388.192	1	0.000	0.101	
Block 3							
gender							
boys (ref.)							
girls	0.067	0.075	0.800	1	0.371	1.070	0.923–1.239
school grade							
grade 7 (ref.)			34.724	2	0.000		
grade 9	–0.243	0.078	9.585	1	0.002	0.785	0.673–0.915
grade 11	–0.591	0.102	33.729	1	0.000	0.554	0.454–0.676
place of residence							
city (ref.)			0.935	2	0.626		
small town	0.056	0.092	0.372	1	0.542	1.058	0.883–1.268
village	–0.018	0.096	0.034	1	0.853	0.982	0.815–1.185
family affluence							
poor (ref.)			0.537	2	0.764		
average	–0.039	0.078	0.254	1	0.614	0.962	0.826–1.120
affluent	–0.074	0.106	0.486	1	0.486	0.928	0.754–1.144
declared time spent on social media							
0–1 h/day (ref.)			136.873	2	0.000		
2–4 h/day	0.448	0.096	21.565	1	0.000	1.564	1.295–1.890
≥5 h/day	1.129	0.102	122.485	1	0.000	3.092	2.532–3.777
health literacy							
low	0.444	0.079	31.744	1	0.000	1.559	1.336–1.819
average (ref.)			34.823	2	0.000		
high	–0.082	0.132	0.384	1	0.536	0.921	0.711–1.194
future orientation							
low (ref.)			84.844	2	0.000		
average	–0.601	0.079	57.456	1	0.000	0.548	0.469–0.640
high	–0.906	0.112	65.287	1	0.000	0.404	0.324–0.503
school achievements							
good (ref.)			2.083	2	0.353		
average and below	0.106	0.079	1.789	1	0.181	1.112	0.952–1.299
very good	0.117	0.112	1.088	1	0.297	1.124	0.902–1.401
constant	–2.039	0.146	194.728	1	0.000	0.130	
Block 4							
gender							
boys (ref.)							
girls	–0.274	0.081	11.476	1	0.001	0.761	0.649–0.891
school grade							
grade 7 (ref.)			45.988	2	0.000		
grade 9	–0.313	0.080	15.397	1	0.000	0.731	0.625–0.855
grade 11	–0.684	0.103	43.689	1	0.000	0.505	0.412–0.618
place of residence							
city (ref.)			0.228	2	0.892		
small town	0.037	0.094	0.157	1	0.692	1.038	0.864–1.247
village	0.005	0.097	0.002	1	0.960	1.005	0.831–1.215
family affluence							
poor (ref.)			0.496	2	0.780		
average	–0.021	0.079	0.073	1	0.787	0.979	0.839–1.143
affluent	–0.076	0.108	0.495	1	0.482	0.927	0.749–1.146
declared time spent on social media							
0–1 h/day (ref.)			97.560	2	0.000		
2–4 h/day	0.385	0.098	15.491	1	0.000	1.469	1.213–1.779
≥5 h/day	0.967	0.103	87.405	1	0.000	2.631	2.148–3.223
health literacy							
low	0.414	0.080	26.972	1	0.000	1.513	1.294–1.770
average (ref.)			31.533	2	0.000		
high	–0.144	0.134	1.147	1	0.284	0.866	0.666–1.127
future orientation							
low (ref.)			55.141	2	0.000		
average	–0.513	0.081	40.136	1	0.000	0.598	0.511–0.701
high	–0.725	0.114	40.112	1	0.000	0.484	0.387–0.606
school achievements							
average and below	–0.033	0.081	0.162	1	0.687	0.968	0.825–1.135
good (ref.)			2.788	2	0.248		
very good	0.151	0.114	1.743	1	0.187	1.163	0.929–1.455
SCL							
0–1 (ref.)							
≥2	0.848	0.095	80.203	1	0.000	2.335	1.939–2.811
WHO-5							
good mood (ref.)			10.344	2	0.006		
reduced mood	0.285	0.094	9.098	1	0.003	1.330	1.105–1.600
at risk of depression	0.283	0.103	7.516	1	0.006	1.327	1.084–1.625
school stress							
low	–0.180	0.101	3.169	1	0.075	0.835	0.685–1.018
average (ref.)			24.035	2	0.000		
high	0.324	0.083	15.357	1	0.000	1.383	1.176–1.627
constant	–2.547	0.166	235.301	1	0.000	0.078	

B – unstandardized regression coefficient; df – degrees of freedom; OR – odds ratio; SE – standard error; Wald – Wald statistic.

Other abbreviations as in Table 2.

Block 5 presented in Table 4 as final model.

In the second block, the time spent on social media was added. Greater time spent on social media increased the risk of PSMU (compared to individuals who spent a maximum of 1 h/day on social media, for 2–4 h/day, the risk increased by 62.6%, and for ≥5 h/day by 273.9%). Introducing this variable resulted in the loss of significance of the gender variable while the importance of age increased.

The third block included individual resource variables. Future orientation emerged as a protective factor – the higher the future orientation, the lower the risk of PSMU. Regarding health literacy, only low HL level was significantly correlated with a higher incidence of PSMU. School achievement was not significantly related to PSMU. The introduction of new variables did not affect the significance of variables from the earlier blocks.

All well-being variables introduced in block 4 were significant. After adding this block, gender became a significant factor again, however, with a reversed effect compared to block 1. Experiencing multiple health complaints was a very important predictor increasing the severity of PSMU by 133.5%. Lowered mood resulted in a 33% higher probability of PSMU, and high school stress increased it by 38.3%. Low school stress reduced the probability of PSMU by 16.5%, but this effect was marginally significant (p < 0.1).

In the fifth block social environment variables were added. The risk of PSMU among students, in the final logistic regression model (Table 4), was most strongly associated with the time spent on social media and the number of health complaints. Higher future orientation, better relationships with parents, and older age were the strongest protective factors. Girls were 23.6% less likely to exhibit PSMU than boys. The addition of social variables reinforced the significance of school stress, which remained positively associated with PSMU. Relationships with peers showed a less clear effect: both below- and above-average peer relationships significantly influenced the model. The supportive school climate was associated with a higher PSMU score, whereas an unfavourable school climate did not differ significantly from the average. Low HL level remained a significant risk factor; high HL levels were not significantly associated. Compared to students with normal mood, those with low mood had a significantly higher risk, while students at risk of depression did not differ significantly from those with normal mood.

**Table 4:** Final logistic regression model of the determinants of problematic social media use (PSMU) (N = 8021), 2024, Poland

Determinant	B	SE	Wald	df	p	OR	95% CI OR
Demographic							
gender							
boys (ref.)						1.000	
girls	–0.269	0.082	10.690	1	0.001	0.764	0.650–0.898
school grade							
grade 7 (ref.)			43.978	2	0.000		
grade 9	–0.318	0.081	15.364	1	0.000	0.728	0.621–0.853
grade 11	–0.672	0.104	41.504	1	0.000	0.510	0.416–0.626
place of residence							
city (ref.)			0.215	2	0.898		
small town	0.036	0.095	0.149	1	0.700	1.037	0.862–1.248
village	0.005	0.098	0.003	1	0.960	1.005	0.829–1.218
family affluence							
low (ref.)			0.133	2	0.936		
average	0.029	0.080	0.130	1	0.718	1.029	0.880–1.204
high	0.022	0.110	0.041	1	0.839	1.023	0.824–1.269
declared time spent on social media							
0–1 h/day (ref.)			100.928	2	0.000		
2–4 h/day	0.402	0.099	16.556	1	0.000	1.495	1.232–1.814
≥5 h/day	1.002	0.105	90.754	1	0.000	2.724	2.216–3.347
Individual resources							
health literacy							
low	0.383	0.081	22.338	1	0.000	1.467	1.251–1.719
average (ref.)			26.256	2	0.000		
high	–0.140	0.136	1.059	1	0.303	0.869	0.666–1.135
future orientation							
low (ref.)			46.350	2	0.000		
average	–0.482	0.082	34.370	1	0.000	0.618	0.526–0.726
high	–0.673	0.116	33.396	1	0.000	0.510	0.406–0.641
school achievements							
good (ref.)			2.212	2	0.331		
average or below	–0.056	0.083	0.460	1	0.498	0.946	0.804–1.112
very good	0.109	0.115	0.894	1	0.344	1.115	0.889–1.399
Well-being indicators							
SCL							
0–1 (ref.)						1.000	
≥2	0.791	0.096	67.473	1	0.000	2.205	1.826–2.663
WHO-5							
good mood (ref.)			5.607	2	0.061		
reduced mood	0.229	0.097	5.562	1	0.018	1.258	1.039–1.521
at risk of depression	0.145	0.110	1.750	1	0.186	1.156	0.932–1.434
school stress							
low	–0.215	0.103	4.371	1	0.037	0.807	0.660–0.987
average (ref.)			23.606	2	0.000		
high	0.315	0.085	13.741	1	0.000	1.370	1.160–1.618
Social environment and support							
parents relationships							
poor	0.445	0.083	28.363	1	0.000	1.560	1.324–1.837
average (ref.)			64.870	2	0.000		
good	–0.565	0.117	23.271	1	0.000	0.568	0.452–0.715
school climate							
unfavorable	0.019	0.085	0.047	1	0.828	1.019	0.862–1.204
average (ref.)			15.702	2	0.000		
supportive	0.375	0.098	14.776	1	0.000	1.456	1.202–1.763
peers and social support							
low	0.304	0.087	12.347	1	0.000	1.355	1.144–1.606
average (ref.)			16.352	2	0.000		
high	0.280	0.096	8.439	1	0.004	1.323	1.095–1.597
Constant	–2.758	0.179	238.555	1	0.000	0.063	

Abbreviations as in Table 2.

Cox-Snell R^2^ = 0.085; Nagelkerke's R^2^ = 0.162.

**Figure 1.**
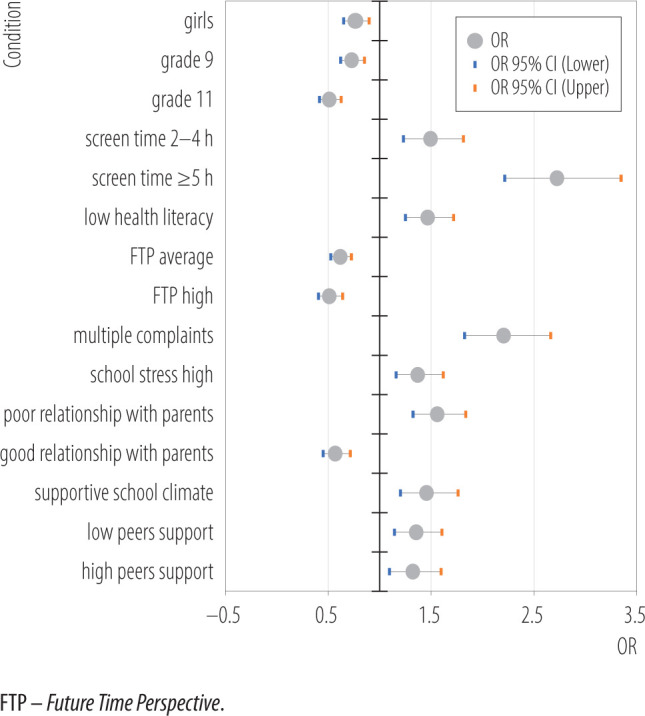
Final logistic model of the relevant conditions of problematic social media use, 2024, Poland

In the final model (Figure 1), place of residence (N = 8021), family affluence and school achievements were not significant. Nagelkerke's R² increased with each block added (R²_1_ = 0.015, R²_2_ = 0.062, R²_3_ = 0.099, R²_4_ = 0.141, R²_5_ = 0.162).

## DISCUSSION

In this study, the determinants of PSMU among Polish adolescents were analysed, taking into account the time spent on social media, socio-demographic and health factors, personal competences as well as social environment, and the findings were related to the results of previous studies. The logistic regression model took into account 14 factors that may affect the risk of PSMU, and for 8 factors the relationship was confirmed. The lack of a relationship with place of residence and family affluence is consistent with the univariate analysis. Two factors relevant to the univariate analysis (school achievements and WHO-5) did not qualify for the final regression model. Relevant predictors can be divided into demographic factors and other risk and protective factors.

### Sociodemographic factors

As expected, age and gender were important predictors. Younger teenagers in the Lubuskie Voivodeship showed higher levels of PSMU compared to older ones, which is consistent with published meta-analyses [19]. The decline in PSMU with age suggests that problematic media use may be related to the stage of adolescent development, including strong dependence on the peer group and immature regulatory mechanisms [20], which may increase vulnerability to PSMU [3]. That behaviour patterns may not necessarily be consolidated in the future [21]. However, individual studies indicate that social media activity increases during adolescence and may not peak until the end of adolescence or early adulthood, suggesting the existence of broader developmental patterns in online behaviour [22].

In the univariate analysis, a higher risk of PSMU was observed among girls [23,24]. Girls are more likely than boys to communicate with friends and others through the internet, which makes them more vulnerable to the symptoms described by the dependent variable [10]. However, in the regression model, the initially statistically significant association between PSMU and gender disappeared after the inclusion of time spent on social media and indices of individual resources, suggesting a suppression effect, whereby these variables accounted for variance that had previously been attributed to gender. Notably, when wellbeing-related variables were introduced in block 4, the association between PSMU and gender re-emerged, this time with the opposite sign. This pattern is consistent with a suppression mechanism, in which controlling for additional variables reveals a relationship that is otherwise obscured or distorted. Ultimately, the results indicate that, after accounting for these factors the boys who were more likely to experience PSMU, which is a novel finding.

### Risk factors

In the model, a higher risk of PSMU was associated with time spent on social media, multiple health complaints, low health literacy, severe school stress, and poorer relationships with parents. A non-intuitive positive relationship was found between PSMU and peer support as well as supportive school climate.

This study showed that PSMU levels were most strongly associated with time spent on social media. Increased exposure (time spent) increases the risk of negative psychological symptoms [23]. However, the latest evidence indicates that risk is not solely a function of duration, but also of platform characteristics, patterns of engagement and individual susceptibility [25].

Among the health and well-being indicators, psychosomatic complaints emerged as the strongest correlate. Previous studies consistently show a strong association between PSMU and adolescents' health complaints. Physical symptoms such as neck and shoulder pain intensified with social media use, especially among girls [26]. It has been demonstrated that individuals who use social media in a problematic way are 1.84 times more likely to report multiple somatic symptoms and 2.60 times more likely to report psychological symptoms [27]. Problematic social media use among adolescents is associated with sleep disorders. Evening use, in particular, leads to exposure to blue light, which causes difficulty falling asleep and, consequently, shorter and poorer-quality sleep [28].

Some symptoms captured by the HBSC-SCL scale such as reduced mood, nervousness and problems falling asleep may also be related to depression. Although earlier studies found that adolescents at risk of PSMU reported high levels of depressive symptoms and increased social media use [29] the authors' final model did not confirm a complete association between PSMU and the risk of depression as measured by WHO-5. Only reduced mood remain significant. However, it is worth noting, that depression risk was significantly associated with the dependent variable before variables related to social relationships were added to the model (Table 3). The authors' results are closer to those of a meta-analysis showing a significant but only minor correlation between adolescents' social media use and depressive symptoms [30].

Previous research suggests that the relationship between school stress and PSMU has been examined primarily in East Asian countries, which may be due to the cultural emphasis on academic achievement [31]. The authors' study showed that strong school stress as well as generalized stress exacerbates PSMU [25].

Univariate analyses confirmed a negative correlation between school climate and problematic smartphone and social media use [32]. However, after adjusting covariates in the logistic regression model, only a supportive school climate was significantly and positively associated with PSMU. Although supportive school climate may be characterized by more flexible behavioural regulations, such as permission to use smartphones on school premises, and stronger peer bonds, the observed shift in the association suggests a suppression effect. Since school climate was introduced in the final block alongside parental and peer support, the high shared variance among these social resources makes it challenging to isolate the specific suppressor. Nevertheless, this may indicate that the protective nature of school climate is complexly intertwined with other social factors.

According to the authors' results, both poor and intensive peer relationships can increase the risk of PSMU. Feelings of loneliness may increase time spent online [25]. The paradoxical increase in PSMU in the context of high social support might be related to intensive communication via messaging apps and the fear of missing out [19].

Large-scale research confirms the authors' finding that the risk of problematic use increases as health knowledge decreases [33]. Frequent exposure to inaccurate yet engaging content may exacerbate unfavorable health behaviours, particularly among individuals with low health literacy.

### Protective factors

The higher the personal competences, the lower the likelihood of PSMU. The protective factors confirmed by the logistic regression model were high future orientation and good relationships with parents.

Particularly large differences in the severity of PSMU were observed depending on future orientation. This topic has not been extensively examined in the existing literature. In the authors' study, the more individuals were able to delay a reward and focus on fulfilling commitments and achieving set goals, the less frequently they exhibited PSMU. Similar associations were reported by Przepiórka and Blachnio [28].

In the literature, a supportive family environment is consistently associated with lower levels of PSMU. Demonstrating affection, being responsive, and establishing clear, proactive rules by parents [34] act as protective factors, whereas inconsistent, or overprotective parenting, family conflicts, and parents ignoring their children in favor of using their own devices (phubbing) increase the risk [35]. Trust and open communication with parents promote responsible social media use, whereas lack of trust or excessive control may lead adolescents to hide information and engage in more risky behaviours [36].

### Strengths and limitations

The conducted study has several important strengths. The most important are its timeliness and broad population coverage – 30% of students in the region participated. Comparative analyses conducted between this study regional data and the nationwide Polish HBSC 2022 sample did not reveal any statistically significant differences on PSMU [15], which increases the representativeness of the findings. The use of standardized measurement tools ensured high reliability and made it possible to compare the results with other studies on similar topics. Including diverse groups of predictors provided a more comprehensive picture of the factors determining PSMU. The obtained R^2^ value is moderately low but as is typical in social research it indicates that the authors' model has captured a significant aspect of this complex phenomenon, which is of great importance both theoretically and practically.

However, several limitations should also be acknowledged. The cross-sectional design does not allow for a deeper understanding of the observed relationships or changes over time. Replication of the study in samples of Polish adolescents would allow population-level trends to be monitored. A more extensive longitudinal study design would help to determine whether, and in what ways, excessive social media use affects functioning in adulthood. Additionally, the use of self-report measures is associated with a potential risk of subjective bias in participants' responses, although this risk is reduced through the use of validated tools. It should also be noted that the analysis did not include all possible determinants that may be relevant for shaping the multidimensional phenomenon. The model's ability to explain 16.2% of the variance highlights the need to continue searching for additional predictors.

## CONCLUSIONS

The authors' research shows, that the PSMU determinants are multidimensional; even when many predictors were considered, the model fit remained modest. This study's findings regarding boys' greater susceptibility to PSMU indicate that health related behaviours are difficult to predict based solely on socio-demographic data (very low R^2^) and a limited set of factors, given the complex nature of these behaviours.

The authors' results confirm the importance of the relational dimension of adolescents' functioning within their peer and family environments. Since time spent on social media shows the strongest correlation with problematic use, the first step should be to intensify efforts to reduce adolescents' smartphone exposure. Physical separation from devices – even through administrative bans such as those implemented in 2025 in Australia – offers a chance for digital detox and encourages engagement in alternative activities that may prove equally rewarding. Limiting the time spent on social media can also alleviate health complaints that are often the result of physical inactivity while using electronic devices.

In addition to the widely discussed risk factors, attention was drawn to the significance of protective factors such as good relationships with parents and the often overlooked construct of future orientation. Improving HL helps prevent addiction-like usage patterns, e.g., by supporting behavioural self-regulation and a critical evaluation of online content. It is worth expanding research on other constructive and beneficial factors that can support such behaviours in the future, especially since social media will remain an integral part of young people's lives.
